# Genome-Wide Identification of the *TCP* Gene Family in *Chimonanthus praecox* and Functional Analysis of *CpTCP2* Regulating Leaf Development and Flowering in Transgenic *Arabidopsis*

**DOI:** 10.3390/plants14193039

**Published:** 2025-10-01

**Authors:** Yinzhu Cao, Gangyu Guo, Huafeng Wu, Xia Wang, Bin Liu, Ximeng Yang, Qianli Dai, Hengxing Zhu, Min Lu, Haoxiang Zhu, Zheng Li, Chunlian Jin, Shenchong Li, Shunzhao Sui

**Affiliations:** 1 Key Laboratory of Agricultural Biosafety and Green Production of Upper Yangtze River (Ministry of Education), Chongqing Engineering Research Center for Floriculture, College of Horticulture and Landscape Architecture, Southwest University, Chongqing 400715, China; yinzhu202108@163.com (Y.C.); 13610126159@163.com (G.G.); swuwhf@126.com (H.W.); 15705983137@163.com (B.L.); zzmm8862024@163.com (Z.L.); 2 Chongqing Key Laboratory of Forest Ecological Restoration and Utilization in the Three Gorges Reservoir Area, Chongqing Academy of Forestry, Chongqing 400036, China; xm2020@email.swu.edu.cn (X.Y.); daiqianli126@126.com (Q.D.); 15213188486@163.com (H.Z.); yimi999@foxmail.com (M.L.); 3 College of Forestry, Gansu Agricultural University, Lanzhou 730070, China; wx221069@163.com; 4 College of Horticulture and Landscape Architecture, Southwest University, Chongqing 400715, China; zhuhx8910@swu.edu.cn; 5 Key Laboratory for Flower Breeding of Yunnan Province, National Engineering Research Center for Ornamental Horticulture, Floriculture Research Institute, Yunnan Academy of Agricultural Sciences, Kunming 650205, China; jinchunlianhh@foxmail.com

**Keywords:** *Chimonanthus praecox*, *TCP* family, *CpTCP2*, leaf development, flowering

## Abstract

TCP transcription factors represent a crucial family of plant regulators that contribute significantly to growth and developmental processes. Although the *TCP* gene family has been extensively studied in various plant species, research on *Chimonanthus praecox* (wintersweet) remains limited. Here, we performed genome-wide identification and analysis of the *TCP* gene family in *C. praecox* and identified 22 *CpTCP* genes. We further systematically examined the associated physicochemical properties, evolutionary relationships, gene structures, and regulatory features. Analysis revealed that all CpTCP proteins possess a conserved TCP domain, and subcellular localization prediction indicated their localization in the nucleus. Promoter analysis revealed that multiple cis-elements are associated with abiotic stress responses and plant growth regulation. Further analysis revealed high *CpTCP2* expression in the leaves and stamen, with significantly increased levels during flower senescence. *CpTCP2* expression was upregulated in response to methyl jasmonate (MeJA), salicylic acid, abscisic acid, and shade. *CpTCP2* overexpression in *Arabidopsis thaliana* resulted in a reduced leaf area, delayed flowering, and increased rosette leaf numbers. Moreover, MeJA treatment accelerated leaf senescence in *CpTCP2* transgenic *Arabidopsis*. These findings provide insights into the evolutionary characteristics of the TCP family in *C. praecox*, highlighting the functional role of *CpTCP2* in regulating leaf development and flowering time in *Arabidopsis*, thereby offering valuable genetic resources for wintersweet molecular breeding.

## 1. Introduction

*Chimonanthus praecox*, commonly known as wintersweet, is a deciduous shrub native to China, renowned for its fragrant yellow flowers that bloom during the cold winter months [1]. Research on this species predominantly focuses on its horticultural value and molecular genetics. Widely cultivated across China, *C. praecox* is frequently used in landscaping, in garden decoration, and as an ornamental shrub, appreciated for its unique flowering season and elegant fragrance [2]. Molecular studies of *C. praecox* primarily explore its genomics [2,3,4], transcription factors [5,6,7,8], and their roles in regulating plant development [9,10,11,12]. Research on the flowering and leaf development of *C*. *praecox* using molecular biology techniques holds practical value.

TCP transcription factors are involved in multiple aspects of plant development, including embryogenesis, leaf development, branching, floral organ morphogenesis, pollen development, germination, senescence, circadian rhythms, cell cycle regulation, hormone signal transduction, and the regulation of cell differentiation, proliferation, and growth [13]. This family was originally named after three transcription factors: *TB1* (Teosinte Branched1) from *Zea mays* [14], *CYC* (Cycloidea) from *Antirrhinum majus* [15], and *PCF* (Proliferating Cell Factor) from *Oryza sativa* [16]. Evolutionary studies have suggested that the TCP family originated from an unknown ancestor that appeared after the emergence of green algae but before terrestrial plants [17]. These proteins contain a highly conserved TCP domain, which is classified into two classes based on the characteristics within and outside the domain, specifically Class I (PCF) and Class II [18]. Class II TCPs have four additional amino acid residues in the conserved base region compared to Class I. Moreover, Class II TCPs are further divided into CIN (Cincinnata) and CYC/TB1 subfamilies [13]. Preliminary studies have suggested that Class I members in *Arabidopsis thaliana* and *Oryza sativa* promote cell proliferation, whereas Class II members, such as *CYC*, *CIN*, and *TB1*, generally inhibit cell proliferation [13].

The CIN subfamily of TCPs plays a central role in regulating leaf size and shape. Mutations in the *CIN* gene of *Antirrhinum majus* result in larger leaves with wavy edges [19]. In *A. thaliana*, *CIN* TCPs exhibit redundant and additive effects on the regulation of leaf morphology. An *Arabidopsis tcp4* mutant shows wavy leaf margins, whereas *tcp4* and *tcp2* double mutants produce larger curled leaves [20]. Further, overexpression of the *TCP4* gene in *Arabidopsis* leads to plants with smaller leaf areas than the wild-type, and overexpressing plants exhibit lanceolate-shaped leaves [21]. Meanwhile, overexpression of the *TCP2* gene in *Arabidopsis* causes the leaves to become outwardly angled and exhibit a greater degree of downward bending [22]. In *Brassica rapa* ssp., the *CIN*-like gene *BrrTCP2* is conserved and controls leaf size and shape. *BrrTCP2* overexpression reduces the leaf size in wild-type *Arabidopsis* and restores the leaf morphology in *tcp2tcp4tcp10* mutants [23]. *CIN*-like TCPs inhibit the activity of the marginal meristem, which determines the complexity of leaf margins or compound leaf formation in different plants. In the Class I *TCP* gene family, *AtTCP7*, *AtTCP8*, *AtTCP22*, and *AtTCP23* exhibit functional redundancies. In *AtTCP7*, *AtTCP8*, *AtTCP22*, and *AtTCP23* multiple mutants, leaf shape and size are similar to those of wild-type *Arabidopsis*. However, when a repression domain is introduced, single mutants of *AtTCP7* or *AtTCP23* exhibit curled rosette leaves and smaller leaf sizes [24]. The *Arabidopsis* KNOX1 (Class I KNOTTED-like homeobox) family is involved in leaf development and compound leaf formation. AtTCP7, AtTCP8, AtTCP22, and AtTCP23 bind directly to the promoter of *STM*, a gene in the *KNOX1* family, suggesting that Class I *TCP* genes in *Arabidopsis* directly or indirectly regulate *KNOX1*-family genes to control leaf development [25].

TCP transcription factors regulate leaf cell growth via lateral and longitudinal cell expansion. Plant hormones, such as ethylene and cytokinins, promote lateral growth, whereas auxins, brassinosteroids, and gibberellins drive longitudinal cell expansion [26]. Studies have shown that reduced expression of the TCP CIN subfamily in *Arabidopsis* results in an increased proportion of smaller cells and a reduced number of cells entering differentiation. In contrast, the overexpression this subfamily results in cell cycle exit in a higher proportion of cells and the induction of cell differentiation, thereby inhibiting further cell division [27]. These findings offer insights into the phenotypes of CIN subfamily mutants, as both loss-of-function and gain-of-function mutations lead to alterations in leaf size and shape.

The TCP family has crucial roles in plant flower development, morphology, and timing. In *Arabidopsis*, an *mir319a* mutant exhibits significant defects in petal development, characterized by the formation of thread-like petals. Meanwhile, the CIN subfamily gene *AtTCP4* restores the mutant phenotype, indicating that *AtTCP4* is a key gene involved in the petal development pathway and regulated by miR319a [28]. In addition, an *spe3* mutant of *Arabidopsis* produces larger petals, and this gene has an opposing function to that of miR319a. *AtSPE3*, an upstream gene, positively regulates the expression of *AtTCP4* and *AtTCP10* and negatively regulates miR319a expression, thereby controlling petal size [29]. Moreover, Class I *TCP* genes regulate flower development. Overexpression of the *PePCF10* gene from *Phalaenopsis aphrodite* in *Arabidopsis* results in smaller petals compared with those in the wild-type, with a reduction in the petal cell area and an increase in the cell number [30]. Genes from the TB1/CYC subfamily also play a role in the regulation of flower development. The overexpression of *Chrysanthemum morifolium Cyc2CL-1* and *Cyc2CL-2* genes in *Arabidopsis* inhibits stamen and petal development [31]. Further, *CmCYC2c* binds to the promoter of *ClCYC2f* to regulate floral symmetry development [32].

In summary, TCP transcription factors are plant-specific and important transcriptional regulators. Although the *TCP* gene family has been extensively studied in many plants, research on the function of this family in *C. praecox* remains relatively scarce. A deeper understanding of the composition, evolutionary characteristics, and functional mechanisms of the *TCP* gene family in *C. praecox* is crucial for elucidating the molecular regulatory networks underlying plant growth and development. Therefore, we aimed to conduct a comprehensive genome-wide identification and systematic analysis of the *TCP* gene family in *C. praecox*, focusing on investigating the functional characteristics of *CpTCP2* to explore its role in growth and development while also enhancing our understanding of the functional diversity of plant TCP transcription factors.

## 2. Results

### 2.1. Identification and Physicochemical Properties of CpTCP Genes

TCP proteins were identified from the *C. praecox* protein database using HMMER software (version 3.0), with the hidden Markov model (PF03634) of TCP as the reference. Domain analysis of the TCP proteins was performed using online tools, such as NCBI-CD Search, Pfam, and SMART, excluding sequences that lacked the characteristic TCP domain. In total, 22 *TCP* family genes from *C. praecox* were identified (Table 1), including Ws006179, Ws001834, Ws008336, Ws004919, Ws020128, Ws013215, Ws002199, Ws000567, Ws026740, Ws018174, Ws021384, Ws013991, Ws025658, Ws007064, Ws004363, Ws000794, Ws005371, Ws022851, Ws007603, Ws017440, Ws002910, and Ws000671.

The physicochemical properties of the identified TCP proteins were analyzed (Table 1). The 22 *C. praecox TCP* genes were named based on their similarity to the 24 *TCP* genes in *A. thaliana*. Amino acid lengths ranged from 209 to 603 aa, the relative molecular weight varied between 22.06 and 65.10 kDa, and the theoretical pI values ranged from 5.90 to 10.20. The instability index ranged from 45.16 to 69.10, and the hydrophobicity index ranged from −0.884 to −0.209. Moreover, the aliphatic indices varied between 58.51 and 79.15. None of the TCP proteins possessed signal peptides or transmembrane domains, and subcellular localization predictions indicated that all TCP proteins are localized to the nucleus.

### 2.2. Multiple Sequence Alignment and Phylogenetic Tree Analysis of CpTCP Proteins

**Figure 1:**
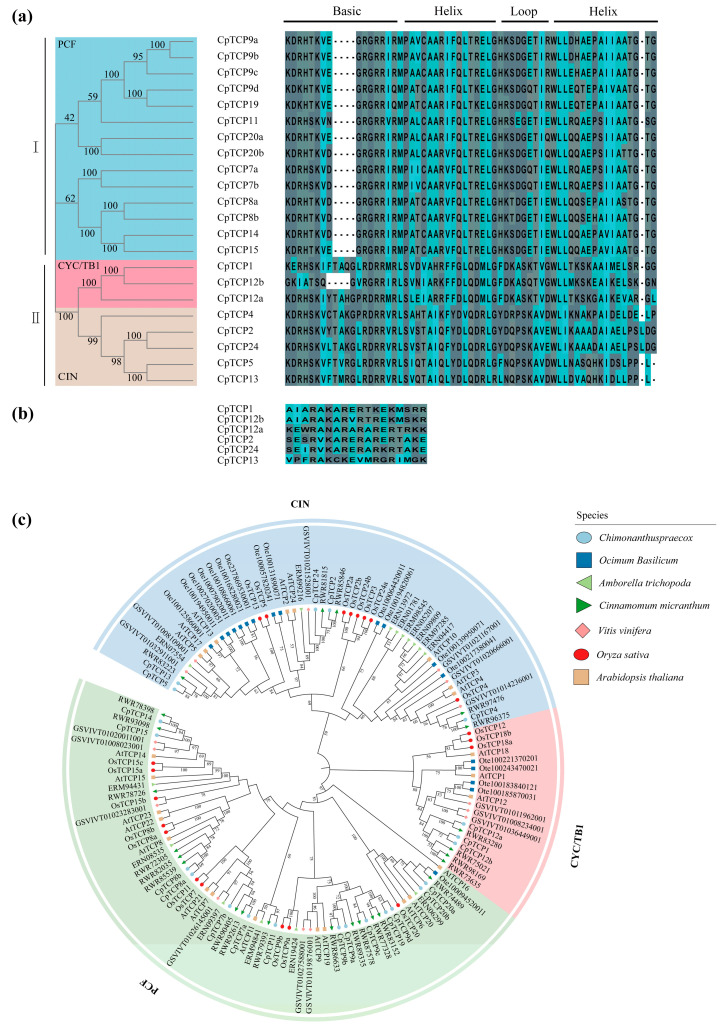
Conserved domain and evolutionary analysis of CpTCP proteins. (**a**) Multiple sequence alignment of CpTCP proteins. (**b**) Alignment of R domains in type II subfamily members. (**c**) Phylogenetic analysis of TCP proteins from *Chimonanthus praecox*, *Arabidopsis thaliana*, *Oryza sativa*, *Ocimum basilicum*, *Vitis vinifera*, *Cinnamomum micranthum*, and *Amborella trichopoda*. The phylogenetic tree was constructed using the neighbor-joining method, with 1000 bootstrap replicates, using MEGA (version 10.0).

Multiple sequence alignment of the conserved TCP domains in the 22 CpTCP proteins revealed two groups: 14 proteins belonging to Class I (PCF) and 8 proteins belonging to Class II. Class II was further divided into the CYC/TB1 and CIN subgroups, with CpTCP1/12a/12b classified in the CYC/TB1 subgroup and CpTCP2/4/24/5/13 classified in the CIN subgroup. The conserved domain of TCP proteins is similar to the bHLH domain. Class II TCP proteins have a 4-amino acid insertion in the basic region compared with those of Class I, although both Class I and II proteins exhibit high amino acid conservation in this region. In the loop and helical regions, amino acid conservation was markedly lower in both classes (Figure 1a). In addition to the TCP domain, 6 Class II CpTCP proteins (CpTCP1/2/12a/12b/13/24) contain an R domain (18 arginine-rich residues) at the C-terminus, which distinguishes classes I and II (Figure 1b). A phylogenetic analysis showed that *C. praecox* TCP proteins cluster closely with those of *Cinnamomum micranthum*, and similar clustering was observed between the Class II subfamily of *C. praecox* and *Vitis vinifera*. No significant clustering was found with *Ocimum basilicum* and *Oryza sativa*, further confirming a close evolutionary relationship between *C. praecox* and dicots, particularly of the Lauraceae family (Figure 1c).

### 2.3. Conserved Motif Analysis and Promoter Cis-Element Analysis of CpTCPs

**Figure 2:**
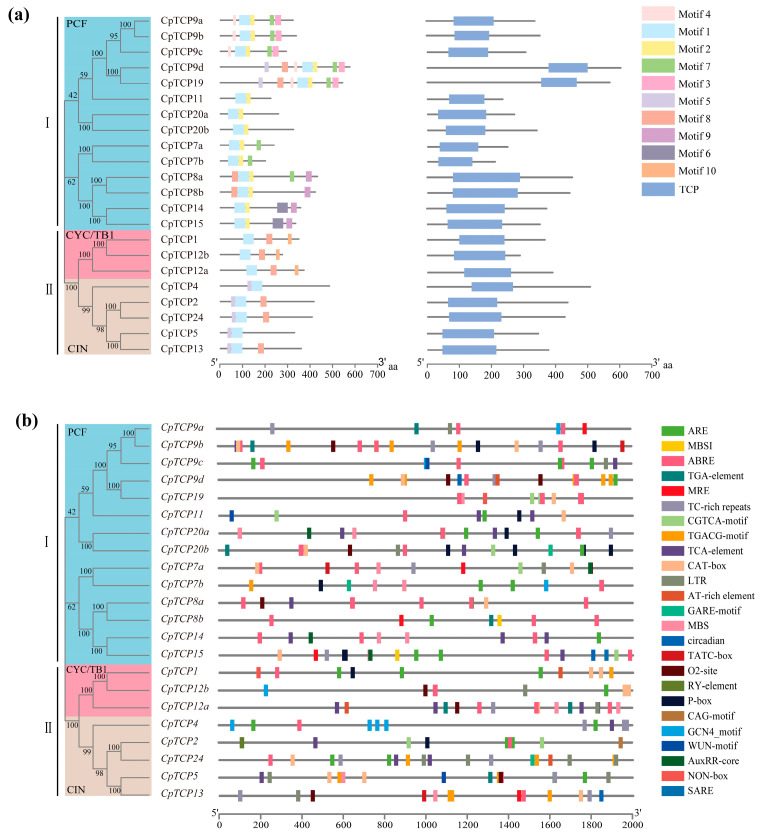
Analysis of conserved motifs and promoter cis-acting elements of CpTCPs. (**a**) Conserved motifs and domains. Motifs 1–10 are represented by differently colored boxes, and the box length represents the motif length. The conserved TCP domain is indicated by a blue box. (**b**) Analysis of *cis*-acting elements in the *CpTCP* promoter.

Conserved motif analysis revealed that CpTCP proteins contain 10 distinct motifs, with the number of motifs per protein ranging from 2 to 7 and all proteins containing Motif1. Results indicated that Class I proteins uniquely harbor Motifs2/3/4/6, whereas the CYC/TB1 subfamily of Class II proteins specifically contains Motif10, which serves as a key marker for distinguishing CYC/TB1 from the other subgroups. Additionally, the CIN subgroup was found to contain Motif5 at the N-terminus, distinguishing it from the CYC/TB1 subgroup. Domain analysis revealed that each CpTCP protein contained a TCP domain. In the Class I subgroups, CpTCP9d and CpTCP19 were determined to have TCP domains closer to the N-terminus (Figure 2a).

The promoter cis-element analysis (Figure 2b) revealed 25 distinct cis-elements in the *CpTCP* family, including elements related to meristem and endosperm expression, defense, and the stress response, as well as abiotic stress-related elements such as those associated with drought, low temperature, and anaerobic induction. Several *CpTCP* promoters were found to contain methyl jasmonate (MeJA)-responsive elements (CGTCA-motif and TGACG-motif), salicylic acid (SA)-responsive elements (TCA-element and SARE), abscisic acid (ABA)-responsive elements (ABREs), gibberellin-responsive elements (GARE-motif, TATC-box, and P-box), and auxin-responsive elements (TGA-element and AuxRR-core). Two cis-acting regulatory elements, CAT-box and NON-box, were determined to be associated with meristem expression during plant growth and development. Multiple *CpTCP* promoters were also found to contain cis-elements involved in endosperm expression (GCN4-motif) and seed-specific regulation (RY element). Thus, C. *praecox CpTCP* genes likely play significant roles in hormone responses, plant growth and development, and stress responses.

### 2.4. Chromosomal Localization and Synteny Analysis of CpTCP Genes

**Figure 3:**
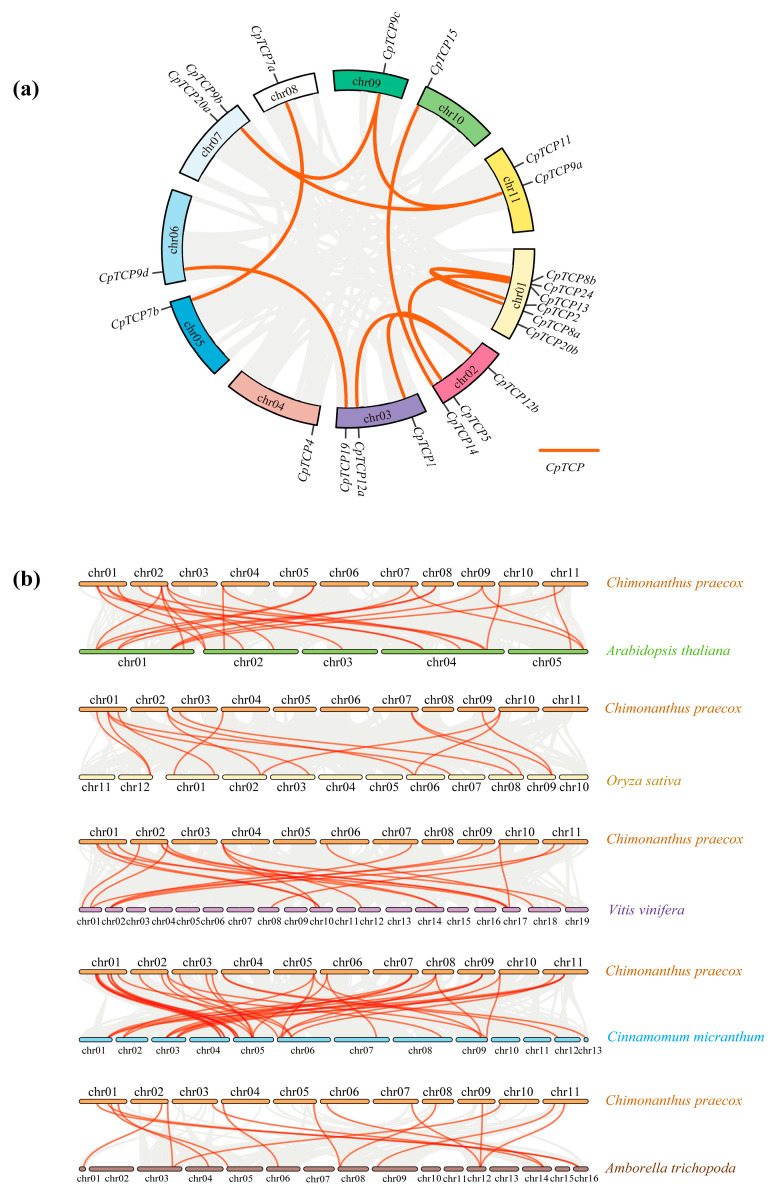
Chromosomal localization and analysis of *CpTCP* family genes. (**a**) Chromosomal locations and synteny of *CpTCP* genes. (**b**) Synteny analysis of *Chimonanthus praecox* with *Arabidopsis thaliana*, *Oryza sativa*, *Vitis vinifera*, *Cinnamomum micranthum*, and *Amborella trichopoda*.

Gene localization analysis showed that *CpTCP* genes were distributed across all chromosomes (Figure 3a). Moreover, synteny analysis of *C. praecox*, *A. thaliana*, *O. sativa*, *V. vinifera*, *Cinnamomum micranthum*, and *Amborella trichopoda* revealed replication events for all coding genes. Eleven pairs of syntenic genes were identified in the *C. praecox* TCP family: *CpTCP8a/8b*, *CpTCP2/24*, *CpTCP5/13*, *CpTCP1/12b*, *CpTCP12a/12b*, *CpTCP14/15*, *CpTCP9d/19*, *CpTCP7a/7b*, *CpTCP9a/9b*, *CpTCP9b/9c*, and *CpTCP9a/9c*. Synteny genes were found on all chromosomes except chromosome 04. Further, *C. praecox* shared 24, 14, 19, 39, and 16 syntenic gene pairs with *A. thaliana*, *O. sativa*, *V. vinifera*, *Cinnamomum micranthum*, and *Amborella trichopoda*, respectively (Figure 3b), indicating a close evolutionary relationship with dicots, particularly of the Lauraceae family. Further analysis showed that *CpTCP2*, *CpTCP4*, *CpTCP9b*, *CpTCP9c*, *CpTCP14*, *CpTCP15*, *CpTCP20b*, and *CpTCP24* exhibit synteny across the five species, suggesting that these genes appeared before the divergence of monocots and dicots and maintained high conservation. Conversely, *CpTCP7b* and *CpTCP20a* were not detected in the synteny analysis, indicating that they may be unique to *C. praecox* or may have emerged at later evolutionary stages.

### 2.5. Analysis of the Sequence Characteristics of CpTCP2

**Figure 4:**
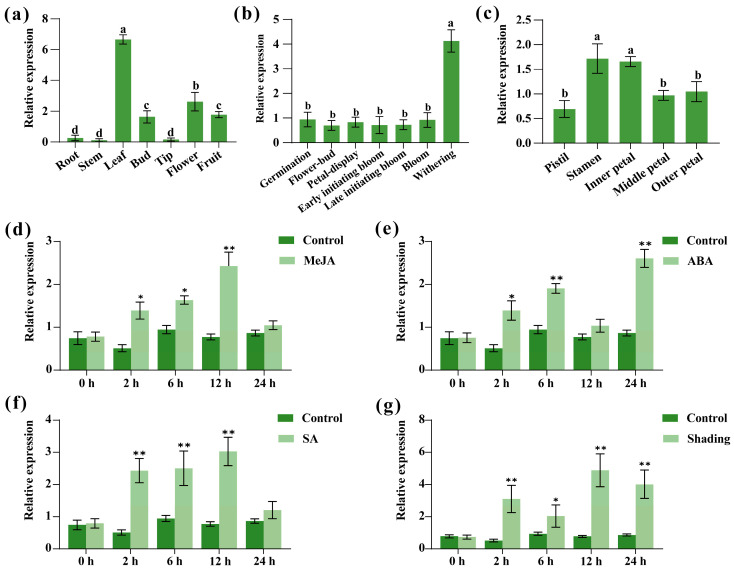
Analysis of the sequence characteristics of *CpTCP2.* Relative expression of *CpTCP2* in the different tissues (**a**), flowering stages (**b**), and floral organs (**c**) of *Chimonanthus praecox.* Different lowercase letters indicate significant differences (*p* < 0.05). Relative expression of *CpTCP2* in response to 100 μmol/L methyl jasmonate (MeJA) (**d**), 50 μmol/L abscisic acid (ABA) (**e**), 50 μmol/L salicylic acid (SA) (**f**), and shading treatment (**g**) in wintersweet leaves. Control refers to leaves collected from untreated wintersweet plants. Asterisks denote statistically significant differences compared with control, * *p* < 0.05, ** *p* < 0.01. The data of each group are presented as the mean ± standard deviation.

Expression analysis revealed that *CpTCP2* was most highly expressed in the leaves of *C. praecox*, which was significantly higher than that in other tissues, and higher expression was also detected in the fruit, flowers, and buds (Figure 4a). The expression of *CpTCP2* was substantially elevated during floral senescence compared with other floral stages, implying that *CpTCP2* plays an important role in the regulation of flower senescence (Figure 4b). Among the floral organs, *CpTCP2* was most highly expressed in the stamen and least expressed in the pistil (Figure 4c).

To explore the effects of exogenous hormones and shade on *CpTCP2* expression, MeJA, ABA, SA, and shade treatments were used to significantly regulate expression (Figure 4d–g). MeJA treatment gradually upregulated *CpTCP2* expression, which peaked at 12 h, whereas ABA treatment caused a gradual increase peaking at 24 h. SA treatment led to the rapid upregulation of *CpTCP2* expression at 12 h, followed by a decrease at 24 h. Shading significantly increased *CpTCP2* expression, which peaked at 12 h. These results suggest that *CpTCP2* regulates senescence in *C. praecox* by influencing plant hormone signaling or photosynthetic processes.

### 2.6. Regulation of Flowering Time and Leaf Development by CpTCP2 in Arabidopsis

**Figure 5:**
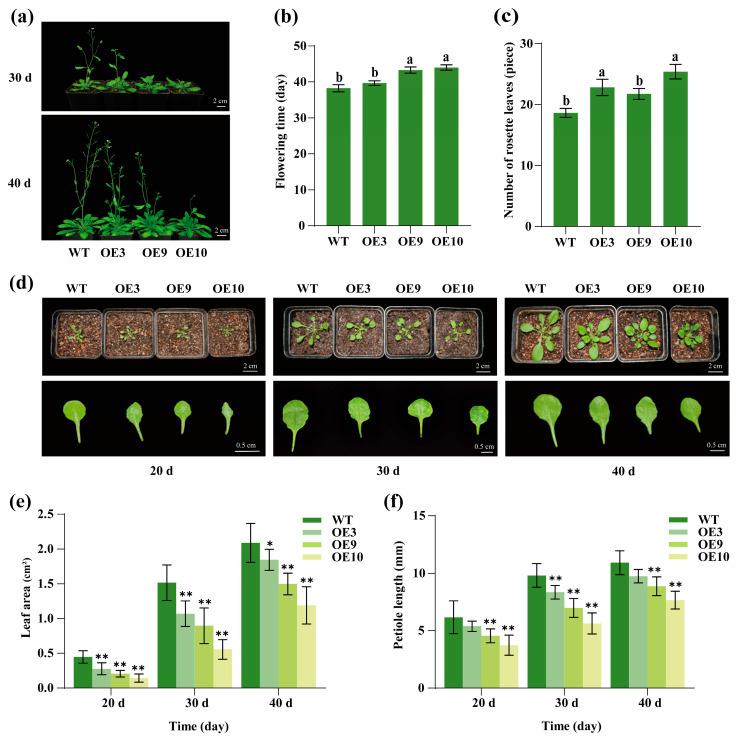
Regulation of flowering time and leaf development by *CpTCP2* in *Arabidopsis.* (**a**) Overexpression of *CpTCP2* delayed flowering in *Arabidopsis thaliana*. Statistical analyses of flowering time (**b**) and the number of leaves produced at the rosette stage of plant growth (**c**) in *CpTCP2*-overexpressing transgenic and WT *Arabidopsis* lines. Different lowercase letters indicate significant differences compared with WT (*p* < 0.05). (**d**) Leaf growth phenotypes of the sixth leaf in *CpTCP2* transgenic and WT *Arabidopsis* lines at 20, 30, and 40 days (bolting shoots were excised at 30 and 40 days). Statistical analyses of leaf area (**e**) and petiole length (**f**) in *CpTCP2* transgenic and WT *Arabidopsis* lines. Asterisks denote statistically significant differences compared with WT, * *p* < 0.05, ** *p* < 0.01. The data of each group are presented as the mean ± standard deviation.

To investigate the functional role of *CpTCP2*, *CpTCP2*-overexpressing transgenic *Arabidopsis* plants were generated by transforming the pCAMBIA1300 vector containing the 35S promoter. Three overexpression lines with low, medium, and high *CpTCP2* expression levels (OE3, OE9, and OE10, respectively) were selected (Figure S1). Phenotypic observations of the T3 generation (Figure 5a) revealed that the OE9 and OE10 lines exhibited significantly delayed flowering compared to the WT, with the differences being statistically significant (Figure 5b). Additionally, the *CpTCP2* overexpression lines produced more rosette leaves than the WT in *Arabidopsis*. (Figure 5c). Measurements of the leaf areas of T3-generation *CpTCP2*-overexpressing lines and the WT showed that those in the OE3, OE9, and OE10 lines were smaller than those of the WT at 20, 30 and 40 days post-sowing, with OE10 having the smallest leaf area (Figure 5d,e). Moreover, the petioles of overexpressing lines were shorter than those of the WT plants (Figure 5f).

**Figure 6:**
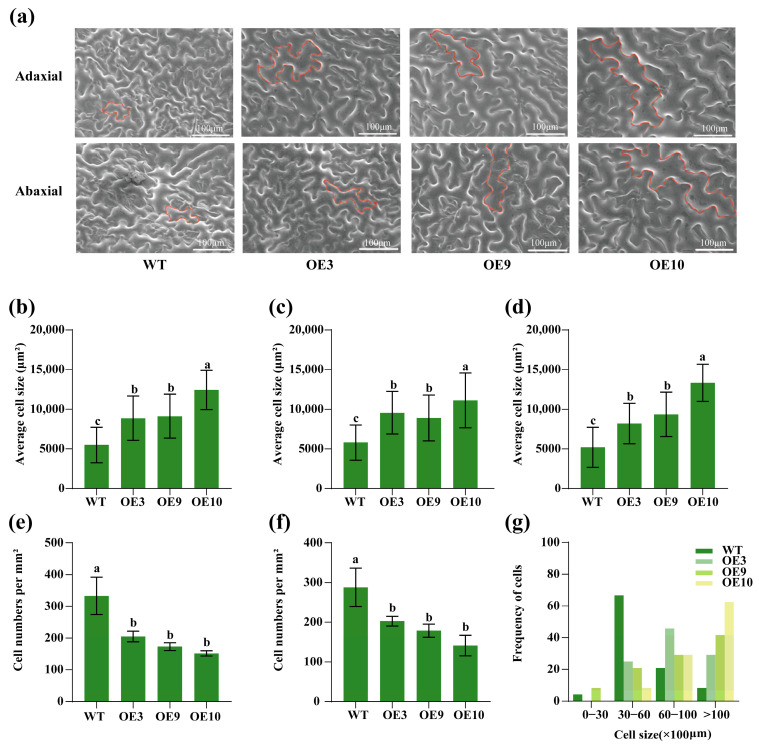
Overexpression of *CpTCP2* increases the leaf cell area and reduces cell numbers in *Arabidopsis thaliana*. Scanning electron microscopy images of leaf epidermal cells (**a**), and statistical analysis of the average cell size (**b**), areas of cells on the adaxial surface (**c**), areas of cells on the abaxial surface (**d**), number of cells on the adaxial surface (**e**), and number of cells on the abaxial surface (**f**). Different lowercase letters indicate significant differences compared with WT (*p* < 0.05). The data of each group are presented as the mean ± standard deviation. (**g**) Distribution of leaf epidermal cell sizes in the different lines. Based on the sixth leaves of *CpTCP2* transgenic and WT *Arabidopsis* plants at 40 days.

Scanning electron microscopy of the sixth leaf epidermal cells revealed that the average cell area and the cell area of both the adaxial and abaxial surfaces were significantly larger in the OE lines compared to those in the WT, with OE10 exhibiting the largest cell area (Figure 6a–d). Additionally, epidermal cell numbers in the leaves of the OE3, OE9, and OE10 lines were significantly reduced compared with the WT, with OE10 exhibiting the lowest level. Further, more than 60% of the cells in the OE10 line had an area greater than 10,000 μm^2^ (Figure 6e–g).

### 2.7. Regulation of Leaf Senescence by CpTCP2 in Arabidopsis

**Figure 7:**
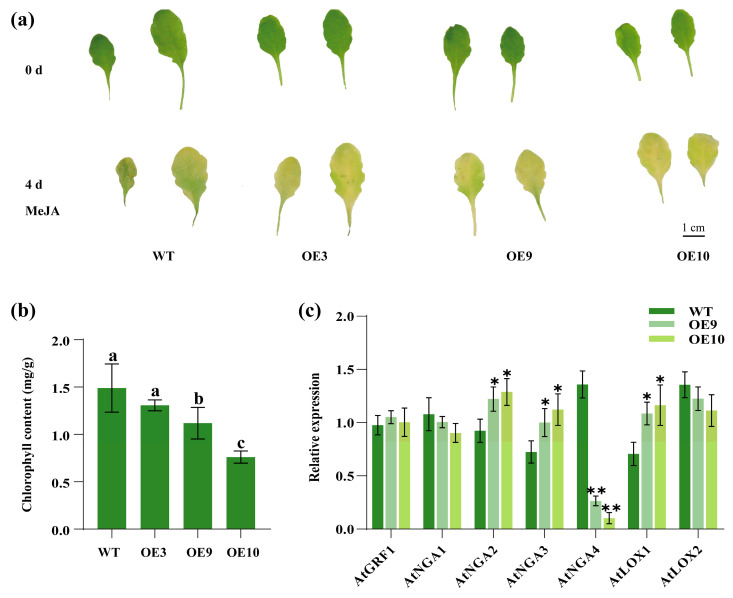
Regulation of leaf senescence by *CpTCP2* in *Arabidopsis thaliana*. Phenotypic observation (**a**) and chlorophyll content (**b**) analysis of *CpTCP2* transgenic and WT *Arabidopsis* leaves 4 days after methyl jasmonate (MeJA) treatment. (**c**) Relative expression levels of endogenous genes in *CpTCP2*-overexpressing and WT *Arabidopsis* lines. The data of each group are presented as the mean ± standard deviation. Asterisks denote statistically significant differences compared with control, * *p* < 0.05, ** *p* < 0.01.

To further explore the regulatory relationship between MeJA and *CpTCP2*, the leaves from *Arabidopsis* plants overexpressing *CpTCP2* were treated with 100 μmol/L MeJA for 4 days (Figure 7a). Both WT and OE lines (OE3, OE9, and OE10) exhibited leaf chlorosis after 4 days of MeJA treatment, with the OE lines showing more pronounced chlorosis compared with WT, and their chlorophyll content was significantly reduced (Figure 7a,b), indicating that *CpTCP2* positively regulates MeJA-induced leaf senescence. To investigate the molecular mechanism through which *CpTCP2* regulates leaf growth and senescence in *C. praecox*, gene expression analysis of the relevant pathways in *Arabidopsis* OE lines (OE10, OE9) was performed. Compared to that in the WT, the expression of *AtNGA2* and *AtNGA3* was significantly upregulated in the OE lines, whereas that of *AtNGA4* was significantly downregulated, and *AtGRF1* and *AtNGA1* showed no significant changes (Figure 7c). These findings suggest that *CpTCP2* regulates leaf development by modulating NGA family members, promoting the expression of *NGA2* and *NGA3,* and inhibiting *NGA4* in *Arabidopsis*. Furthermore, *CpTCP2* may affect JA accumulation by upregulating *AtLOX1* expression, thereby regulating leaf senescence in *Arabidopsis*.

## 3. Discussion

Plant-specific TCP transcription factors play multifaceted regulatory roles in plant growth and development, as demonstrated in various species. In the present study, 22 non-redundant *TCP* genes were identified in *C. praecox*, and they were found to be distributed across 11 chromosomes. Based on their similarity to homologous genes in *A. thaliana*, these genes were named, similar to the naming convention used for the 63 TCP-family genes in *Brassica juncea* [33]. A phylogenetic analysis indicated that these genes could be divided into Classes I and II, with Class II further subdivided into CYC/TB1 and CIN subgroups. This classification is consistent with *TCP* gene families in *Arabidopsis* [34], grape [35], and maize [36]. This classification is consistent with the classification of TCP gene families in other species, showing the conservation of *TCP* gene families across plants.

All CpTCP proteins were determined to contain a conserved TCP domain, and Class II CpTCP proteins were found to lack Motif 2. In *Capsicum annuum*, CaTCPs in the same subgroup exhibit similarities in motif number and distribution [37]. A similar motif composition was observed in *BcTCP* proteins in *Brassica campestris* L. ssp. chinensis, where motifs are clustered within the same subgroup, whereas structural differences between subgroups may reflect functional divergence among them [38]. These structural variations may reflect functional divergence among TCP subgroups, suggesting that even within the same class, the TCP proteins may have evolved distinct roles in plant development and stress responses. Promoter analysis revealed that the *CpTCP* promoters are rich in cis-regulatory elements related to hormone responses, stress, and developmental regulation, suggesting their potential role in various biological processes. The upregulation of *CpTCP2* expression under hormone and shade treatments further suggests that this gene is involved in the regulation of environmental adaptability. Moreover, expression pattern analysis showed that multiple *CpTCP* genes exhibit significant responses to ABA and MeJA treatments, with the expression levels of some genes being rapidly upregulated or downregulated within a short time, indicating their involvement in mediating plant hormone signaling through multiple pathways. In *Dimocarpus longan*, the expression levels of most *DlTCP* genes are significantly downregulated 12 h after ABA induction, and a significant upregulation or downregulation of most *DlTCP* genes is observed at 2 h [39]. It suggests that TCP genes may play a role in the early response of plants to environmental stresses and may be involved in stress adaptation and hormone signaling pathways.

Additionally, the variations in *CpTCP2* expression among different tissues suggest its potential role in organ development. Specifically, *CpTCP2* was expressed at the highest levels in leaves, with the strongest expression in stamen, among floral organs, and expression was significantly elevated during flower senescence. In *Prunus mume*, the gene homologous to *AtTCP2*, *PmTCP09*, shows the highest expression in leaves [40], and in grapes, the homologous gene *VvTCP6* is most highly expressed in young leaves, higher than that in mature and senescent leaves [35]. However, in maize, the expression levels of *ZmTCP17* and *ZmTCP03*, which are homologous to *AtTCP2*, are significantly lower in the leaves than in the stems [36]. This contrast suggests that TCP2 homologs may exhibit tissue-specific expression patterns across species, reflecting functional diversification during evolution. Specifically, TCP2 genes are generally highly expressed in the leaves, which may indicate their involvement in regulating leaf growth and development. These discrepancies highlight the potential for TCP2 genes to play distinct roles in growth and developmental processes in different plant species.

In the present study, *CpTCP2* overexpression in *Arabidopsis* led to significantly smaller leaves compared to those in wild-type plants, with fewer leaf cells and larger cell areas. Previous studies have reported that enhanced expression of CIN-subgroup TCPs leads to more cells exiting the cell cycle and undergoing differentiation, thus inhibiting cell division [27]. Therefore, *CpTCP2* may regulate leaf size and morphology by controlling cell division and differentiation in *Arabidopsis*. Furthermore, *CpTCP2* overexpression promoted MeJA-mediated leaf senescence in *Arabidopsis*, with a more obvious reduction in chlorophyll content compared to that in the wild-type. In *Arabidopsis*, *AtTCP4* overexpression with 60 and 300 μmol/L MeJA treatment resulted in more pronounced leaf senescence compared to that in the wild-type [41]. This suggests the potential of TCP genes in regulating the senescence process through MeJA. Further, in *CpTCP2*-overexpressing *Arabidopsis* lines, expression of the JA biosynthesis-related gene *AtLOX1* was significantly upregulated, whereas *AtLOX2* expression remained unchanged, suggesting that *CpTCP2* influences JA synthesis through the LOX1 pathway, rather than the LOX2 pathway, thereby promoting leaf senescence [42]. In *Arabidopsis*, *AtTCP2* and *AtTCP3* regulate leaf growth and development by promoting the expression of *AtNGA3*, leading to smaller leaf sizes and the inhibition of leaf growth [43]. In the *CpTCP2*-overexpressing *Arabidopsis* lines OE10 and OE9, *AtNGA2* and *AtNGA3* expression was significantly upregulated, whereas that of *AtNGA4* was significantly downregulated. These results suggest that *CpTCP2* regulates leaf growth and development by promoting *NGA2* and *NGA3* expression and inhibiting *NGA4* expression. Additionally, there were no significant changes in *AtGRF1* expression, suggesting that *CpTCP2* might not be involved in the miR396–GRF regulatory pathway [21]. Studying the flowering and leaf development of *C*. *praecox* using molecular biology techniques holds potential value, providing key genetic resources for breeding. This can aid in the development of new wintersweet cultivars and improve their growth characteristics and flowering traits. This study revealed that *CpTCP2* plays a role in the regulation of leaf growth and development; however, the signaling pathways through which *CpTCP2* exerts its function in leaf development remain to be elucidated.

## 4. Materials and Methods

Wintersweet seeds were collected from Southwest University (Chongqing, China). The seeds were immersed in 98% sulfuric acid for 30 min, thoroughly rinsed with running water, surface-sterilized, and sown in pots containing a peat–vermiculite mixture (3:1, *v*/*v*). Plants were cultivated under controlled conditions of 25 °C, 70% relative humidity, 120 µmol·m^−2^·s^−1^ cool white fluorescent light, and a 16/8 h light/dark photoperiod [5].

Wild-type *Arabidopsis* (Columbia ecotype) seeds were obtained from the Flower Laboratory, College of Horticulture and Landscape Architecture, Southwest University (Chongqing, China). Both wild-type and transgenic *Arabidopsis* plants were grown in a peat: vermiculite mixture (1:1, *v*/*v*) under the same plant cultivation conditions described above.

### 4.1. Identification, Multiple Sequence Alignment, and Evolutionary Analysis of the CpTCP Gene Family

Sequences related to *C. praecox* were downloaded from published genome data [3]. Genome, CDS, protein, and annotation information for *A. thaliana* and *Amborella trichopoda* were obtained from the National Center for Biotechnology Information (NCBI) website (https://www.ncbi.nlm.nih.gov, accessed on 12 June 2021)) and Arabidopsis Information Resource (TAIR) database [44], respectively. Genome, CDS, protein, and annotation data for *V. vinifera* and *O. sativa* were downloaded from the Ensembl Plant website (https://plants.ensembl.org/index.html, accessed on 14 June 2021). The protein sequences of the *TCP* gene family in *O. sativa*, *V. vinifera*, *A. trichopoda*, and *O. basilicum* were retrieved from the PlantTFDB website (https://planttfdb.gao-lab.org/, accessed on 12 June 2021).

Local BLAST (version 2.2.31+) and HMMER software (version 3.0) were used to identify TCP family members in *C*. *praecox*. Initially, a local *C*. *praecox* genome database [3] was constructed using BLAST (version 2.2.31+), with the E-value threshold set to 1 × 10^−5^. The 24 TCP proteins from *A*. *thaliana* [44] were aligned against this database to obtain putative *C. praecox* TCP protein sequences, and redundant sequences or those with less than 50% similarity were removed. The corresponding protein sequences were then extracted from the *C. praecox* protein database using the Fasta Extract function in Tbtools (version 1.045) [45]. Subsequently, the Hidden Markov Model (HMM) profile of the TCP domain (PF03634) was downloaded from the Pfam database [46] and used with the Hmmsearch program in HMMER (version 3.0) to perform a genome-wide search of the *C. praecox* protein database. Homologous protein sequences identified in this search were also extracted using TBtools [45]. Domain screening and verification of the alignment results were conducted using the NCBI-CDD (https://pubmed.ncbi.nlm.nih.gov/, accessed on 20 June 2021), Pfam (http://pfam.xfam.org/null, accessed on 20 June 2021), and SMART (http://smart.embl-heidelberg.de/, accessed on 20 June 2021) platforms to remove redundant sequences and those lacking the typical TCP domain. A final list of *C. praecox* TCP family members was obtained. The ExPasy tool (http://web.expasy.org/protparam/, accessed on 15 July 2021) was used to analyze the primary physical properties of the TCPs. Subcellular localization was predicted using Cell-PLoc software (version 2.0; http://www.csbio.sjtu.edu.cn/bioinf/Cell-PLoc-2/, accessed on 15 July 2021).

Multiple sequence alignments of *C. praecox* TCP proteins were performed using the ClustalW algorithm in MEGA (version 10.0) software, and a phylogenetic tree was constructed. A cross-species evolutionary analysis was conducted based on *A. thaliana*, *O. sativa*, *O. basilicum*, *V. vinifera*, *Cinnamomum micranthum*, and *A. trichopoda*. The neighbor-joining method was used, with 1000 bootstrap replicates. The resulting phylogenetic tree was visualized and enhanced using the iTOL platform (https://itol.embl.de/, accessed on 10 January 2022).

### 4.2. Gene Structure, Conserved Motif, and Promoter Element Analysis

The CDS and 2000 bp upstream promoter sequences of the *C. praecox TCP* genes were extracted using TBtools [45]. Conserved protein motifs were analyzed using the MEME Suite online tool (https://meme-suite.org/meme, accessed on 2 July 2021), with the maximum number of motifs set to 10. Conserved domains were identified using the NCBI-CDD platform. Gene structure diagrams, motif distribution maps, and protein domain diagrams were visualized using TBtools [45]. Promoter cis-acting element analysis was performed using the PlantCARE platform (http://bioinformatics.psb.ugent.be/webtools/plantcare/html/, accessed on 2 July 2021) with a focus on the identification of regulatory elements related to hormone responses and stress resistance. These elements were visualized using the TBtools CSimple BioSequence Viewer [45].

### 4.3. Chromosome Localization and Synteny Analysis

Chromosomal location information for *C. praecox TCP* genes was extracted and visualized using TBtools software [45]. The one-step MCScanX tool was used to identify collinear relationships between *C. praecox* and other species, including *A. thaliana*, *O. sativa*, *V. vinifera*, *C. micranthum*, and *A. trichopoda*. Collinearity plots were generated using the Dual Synteny Plot tool. To further investigate the collinearity within the *C. praecox* species, the Ka/Ks values and divergence time (T = Ks/2λ, λ = 3.02 × 10^−9^) were calculated [47]. Chromosomal collinearity maps were generated using Advanced Circos and further refined using Adobe Illustrator 2020 (Adobe Inc., San Jose, CA, USA).

### 4.4. Cloning of CpTCP2 Gene and Arabidopsis Transformation

Total RNA was extracted from *C. praecox* flowers using the EASYspin Plant RNA Extraction Kit (Boer, Beijing, China). Total RNA was extracted from *C. praecox* and *A. thaliana* using the EASYspin Plant RNA Extraction Kit (Boer, Beijing, China). Total RNA integrity was verified by electrophoresis on a 1% agarose gel, which showed three clear bands (28S, 18S, and 5S rRNA) with the 28S band approximately twice as intense as the 18S band. RNA purity was assessed using a NanoDrop spectrophotometer (Thermo Fisher Scientific, Waltham, MA, USA), and samples with OD_260/280_ ratios of 1.8–2.0 were considered suitable for subsequent experiments. cDNA was synthesized using the PrimeScript RT Kit (TaKaRa, Dalian, China). Based on the *CpTCP2* CDS sequence obtained from transcriptome sequencing, specific primers were designed using Primer Premier software (version 5.0) (Table S1). For PCR amplification, cDNA synthesized from wintersweet was used as the template, and reactions were carried out with TransStart^®^ Top taq DNA polymerase (TransGen, Beijing, China) under the following cycling conditions: an initial denaturation at 94 °C for 5 min; 30 cycles of denaturation at 94 °C for 30 s, annealing at 57 °C for 30 s, and extension at 72 °C for 100 s; followed by a final extension at 72 °C for 10 min. The amplified products were subsequently cloned into the pMD19-T vector (Takara, Shiga, Japan) and verified by sequencing.

*CpTCP2* CDS sequence was cloned into the pCAMBIA1300 plant overexpression vector containing the 35S promoter, using *Kpn*I and *Bam*HI as restriction sites. Homologous recombination primers were designed (Table S1), resulting in the successful construction of the 35S::*CpTCP2* expression vector. This vector was introduced into the *Agrobacterium tumefaciens* strain GV3101 (Weidibio, Shanghai, China) via electroporation. The recombinant GV3101 strain carrying the 35S::*CpTCP2* construct was cultured in YEB liquid medium to an OD_600_ = 0.8, followed by transformation into wild-type *Arabidopsis* plants using the floral dip method [48]. T0 seeds were sown on MS medium containing 25 mg/L hygromycin for transgenic selection. After transformation, DNA was extracted from the leaves of both transgenic and wild-type *Arabidopsis* plants. PCR amplification was performed using *CpTCP2*-specific primers (*CpTCP2* F/R) (Table S1), and the PCR products were analyzed by 1% agarose gel electrophoresis, following the PCR program described above. The 35S::*CpTCP2* plasmid was used as the positive control, and wild-type *Arabidopsis* was used as the negative control. Electrophoresis results were used to confirm the successful integration of the *CpTCP2* gene into the genome of the transgenic plants.

For functional analysis, T1 seeds from transgenic T0 plants were selected on MS medium with 25 mg/L hygromycin. After obtaining homozygous lines from the T3 generation, total RNA was extracted and reverse-transcribed into cDNA. The expression levels of *CpTCP2* were quantified by qRT-PCR, with *AtActin* as the internal reference gene [5]. Lines with high, medium, and low expression (OE10, OE9, and OE3, respectively) were selected for further phenotypic analysis.

### 4.5. Phenotypic Observation and Measurements of Transgenic Plants

Twelve plants each from the T3 high-, medium-, and low-expression *Arabidopsis* lines, as well as wild-type plants, were selected for growth measurements. The sixth leaf was marked when it was fully expanded, and samples were collected on days 20, 30, and 40 after sowing. The length, width, and petiole length of the sixth leaf were measured, and the leaf area was calculated using ImageJ software (version 1.53e). The number of rosette leaves and flowering time were recorded when the inflorescence reached 1 cm. The morphology of the epidermal cells on the adaxial and abaxial surfaces of the sixth leaf of both the OE and WT lines was observed using a HITACHI scanning electron microscope (Hitachi, Tokyo, Japan), and the number and area of cells per field were measured using ImageJ. Rosette leaves from both OE and WT lines were treated with a 100 μmol/L MeJA solution and kept in the dark for 4 days, after which photos were taken, and the chlorophyll content was determined.

### 4.6. Gene Expression Analysis

Two-year-old seedlings of the ‘Qingkou’ variety were used to analyze the expression patterns of *CpTCP* genes in *C. praecox*. Samples were collected from the apex, root, stem, leaf, flower, bud, and seed, as well as from different flowering stages and floral organs. At the six-leaf stage, wintersweet plants were subjected to treatments in which leaves were sprayed with 100 μmol/L MeJA, 50 μmol/L ABA, or 50 μmol/L SA or subjected to shading using black plastic bags. The control was defined as samples collected from untreated wintersweet plants. Leaves were harvested at 2, 6, 12, and 24 h post-treatment. Wild-type *A*. *thaliana*. All collected samples were promptly frozen in liquid nitrogen and preserved at −80 °C.

Specific qRT-PCR primers were designed with Primer Premier software (version 5.0) (Table S1), and qRT-PCR was performed using the Bio-Rad CFX96 system. The reaction system was set up according to the instructions of SsoFast™ EvaGreen^®^ Supermix. The qRT-PCR was conducted under the following cycling conditions: an initial denaturation at 95 °C for 3 min, followed by 40 cycles of 95 °C for 5 s, 60 °C for 5 s, and 72 °C for 5 s, and finalized with a melt curve analysis ranging from 65 °C to 95 °C. *AtActin* was used as an internal reference for the normalization of *Arabidopsis* data (Table S1) [5]. *CpActin* were used as internal reference genes for data normalization in wintersweet [9]. Each treatment included three biological and three technical replicates. Data were analyzed using the 2^−ΔΔCT^ method to calculate relative expression levels [49].

### 4.7. Statistical Analysis

Data were analyzed via one-way analysis of variance and Duncan’s test using IBM SPSS Statistics software (version 22) (SPSS, Chicago, IL, USA). Values of *p* < 0.05 and *p* < 0.01 were considered statistically significant and extremely significant, respectively. Graphical representations were generated using GraphPad Prism (version v8.0.1) (GraphPad Software, San Diego, CA, USA).

## 5. Conclusions

In total, 22 *CpTCP* genes were identified in the *C. praecox* genome, including 14 Class I and 8 Class II members, all of which contain the conserved TCP domain. The promoter regions are rich in cis-elements associated with hormone and stress responses, and some members are involved in developmental regulation, suggesting their potential roles in various biological processes. As a representative member of the CIN subfamily, *CpTCP2* exhibited the highest expression in the leaves and strongest expression in the stamen, among the floral organs. Furthermore, its expression was significantly upregulated during flower senescence in response to SA, MeJA, ABA, and shade treatments, indicating its ability to respond to multiple hormones and environmental signals. Overexpression of *CpTCP2* in *Arabidopsis* resulted in reduced leaf area and delayed flowering, and MeJA induction further aggravated leaf yellowing and senescence. These findings suggest that *CpTCP2* may participate in the regulation of leaf development and senescence by modulating cell division and differentiation as well as responding to JA signaling. Future studies should aim to identify the upstream regulators, downstream targets, and protein interaction partners of *CpTCP2* to establish its regulatory network in *C. praecox*. Moreover, *CpTCP2* is expected to be a candidate gene that regulates leaf morphology and senescence, offering potential targets for genetic improvements and the molecular breeding of *C. praecox*.

## Figures and Tables

**Table 1:** The physicochemical properties of CpTCP family proteins in *C. praecox*.

Name	Gene ID	Homolog	AA	MW	pI	II	GRAVY	AI	SP	TH	SL
CpTCP1	Ws006179	AtTCP1	365	40,312.96	6.57	51.63	−0.707	59.42	NO	0	Nucleus
CpTCP2	Ws001834	AtTCP2	436	47,788.71	6.77	45.49	−0.782	58.51	NO	0	Nucleus
CpTCP4	Ws008336	AtTCP4	508	55,927.39	6.38	68.71	−0.884	60.39	NO	0	Nucleus
CpTCP5	Ws004919	AtTCP5	345	38,523.92	8.43	62.36	−0.743	64.99	NO	0	Nucleus
CpTCP7a	Ws020128	AtTCP7	249	25,889.27	9.90	54.46	−0.244	76.59	NO	0	Nucleus
CpTCP7b	Ws013215	AtTCP7	209	22,059.89	9.29	57.09	−0.340	67.42	NO	0	Nucleus
CpTCP8a	Ws002199	AtTCP8	452	47,290.06	7.07	53.85	−0.563	65.95	NO	0	Nucleus
CpTCP8b	Ws000567	AtTCP8	442	46,544.66	7.94	63.48	−0.486	66.33	NO	0	Nucleus
CpTCP9a	Ws026740	AtTCP9	338	35,322.83	9.55	59.55	−0.315	73.11	NO	0	Nucleus
CpTCP9b	Ws018174	AtTCP9	353	36,308.07	10.20	56.89	−0.209	77.76	NO	0	Nucleus
CpTCP9c	Ws021384	AtTCP9	306	32,376.73	9.13	69.60	−0.279	79.15	NO	0	Nucleus
CpTCP9d	Ws013991	AtTCP9	603	65,091.48	5.90	55.38	−0.502	71.38	NO	0	Nucleus
CpTCP11	Ws025658	AtTCP11	234	24,461.38	6.65	56.45	−0.355	63.55	NO	0	Nucleus
CpTCP12a	Ws007064	AtTCP12	389	43,437.09	6.79	45.16	−0.801	59.51	NO	0	Nucleus
CpTCP12b	Ws004363	AtTCP12	288	32,374.64	9.91	52.48	−0.755	65.69	NO	0	Nucleus
CpTCP13	Ws000794	AtTCP13	376	42,297.49	8.99	57.15	−0.700	65.35	NO	0	Nucleus
CpTCP14	Ws005371	AtTCP14	373	40,123.80	7.24	60.09	−0.591	60.72	NO	0	Nucleus
CpTCP15	Ws022851	AtTCP15	350	37,916.24	7.22	51.49	−0.610	63.94	NO	0	Nucleus
CpTCP19	Ws007603	AtTCP19	569	61,907.04	6.35	53.18	−0.543	70.47	NO	0	Nucleus
CpTCP20a	Ws017440	AtTCP20	270	28,772.23	9.13	58.33	−0.554	65.81	NO	0	Nucleus
CpTCP20b	Ws002910	AtTCP20	340	36,678.30	6.83	58.50	−0.426	71.41	NO	0	Nucleus
CpTCP24	Ws000671	AtTCP24	427	46,947.91	8.27	54.99	−0.791	59.72	NO	0	Nucleus

AA, number of amino acids; MW, molecular weight; pI, theoretical isoelectric point; II, instability index; GRAVY, grand average of hydropathicity; AI, aliphatic index; SP, signal peptide; TH, transmembrane helix; SL, subcellular location prediction.

## Data Availability

Data are contained within the article and Supplementary Materials.

## Supplementary Materials

The following supporting information can be downloaded at: https://www.mdpi.com/article/10.3390/plants14193039/s1, Table S1: List of primers; Figure S1: Expression of *CpTCP2* gene in different transgenic *Arabidopsis* lines.
