# Supplementary material for: Genome-Wide Identification of the TCP Gene Family in Chimonanthus praecox and Functional Analysis of CpTCP2 Regulating Leaf Development and Flowering in Transgenic Arabidopsis
Source: Plants (Basel). 2025 Oct 1;14(19):3039. doi: 10.3390/plants14193039 (PMC12526109; doi:10.3390/plants14193039)
Supplement: Supplementary file 1 [file plants-14-03039-s001.zip › Supplementary Materials/Table S1.pdf]

Table S1. List of primers

Primer name	Primer sequences (5′ 3′)
CpTCP2-F	GCACAGTTCATGTCTCAGGCGTGC
CpTCP2-R	CATTAGAGTGTTCTCTACTTGCCG
1300-CpTCP2-F	ACGGGGGACGAGCTCGGTACC<br>ATGGAGATGGATGACATGGAG
1300-CpTCP2-R	CATGTCGACTCTAGAGGATCC<br>GCTTTTTCCTTTTCCCTTCAG
qCpActin-F	AGGCTAAGATTCAAGACAAGG
qCpActin-R	TTGGTCGCAGCTGATTGCTGTG
qCpTCP2-F	ACTGCGATTCAGTTCTACGAT
qCpTCP2-R	CAGCTTCAAAGCGCCTAT
qAtActin-F	CTTCGTCTTCCACTTCAG
qAtActin-R	ATCATACCAGTCTCAACAC
AtNGA1-F	AGACAAAAAGCTTGACGCCG
AtNGA1-R	GCGAAATGAGGATGGTCAGG
AtNGA2-F	CTCATCCTCAGATGCCGACAA
AtNGA2-R	CTTTGCATCATCACCGGCAC
AtNGA3-F	ATGACCAAAGGATGGAGCCG
AtNGA3-R	TTGAACGAGGCTCATGTCGG
AtNGA4-F	TAACGCACAACCGGAGAGAC
AtNGA4-R	GTCGGAGACCCGAAAACGAA
AtGRF1-F	GGACTTCAGCTGTTGCGATG
AtGRF1-R	CGTGGCAGGAAAAACCGAAG
AtLOX1-F	GCCGTTGGTGATAGAGCTGA
AtLOX1-R	CAACTGCCATAGCGAGTCGT
AtLOX2-F	GGACTCATGCCTGTACGGAG
AtLOX2-R	CCGTTGACAAGACTTTGGCG
