# Supplementary figures and images for: Genome-Wide Identification of the TCP Gene Family in Chimonanthus praecox and Functional Analysis of CpTCP2 Regulating Leaf Development and Flowering in Transgenic Arabidopsis
Source: Plants (Basel). 2025 Oct 1;14(19):3039. doi: 10.3390/plants14193039 (PMC12526109; doi:10.3390/plants14193039)

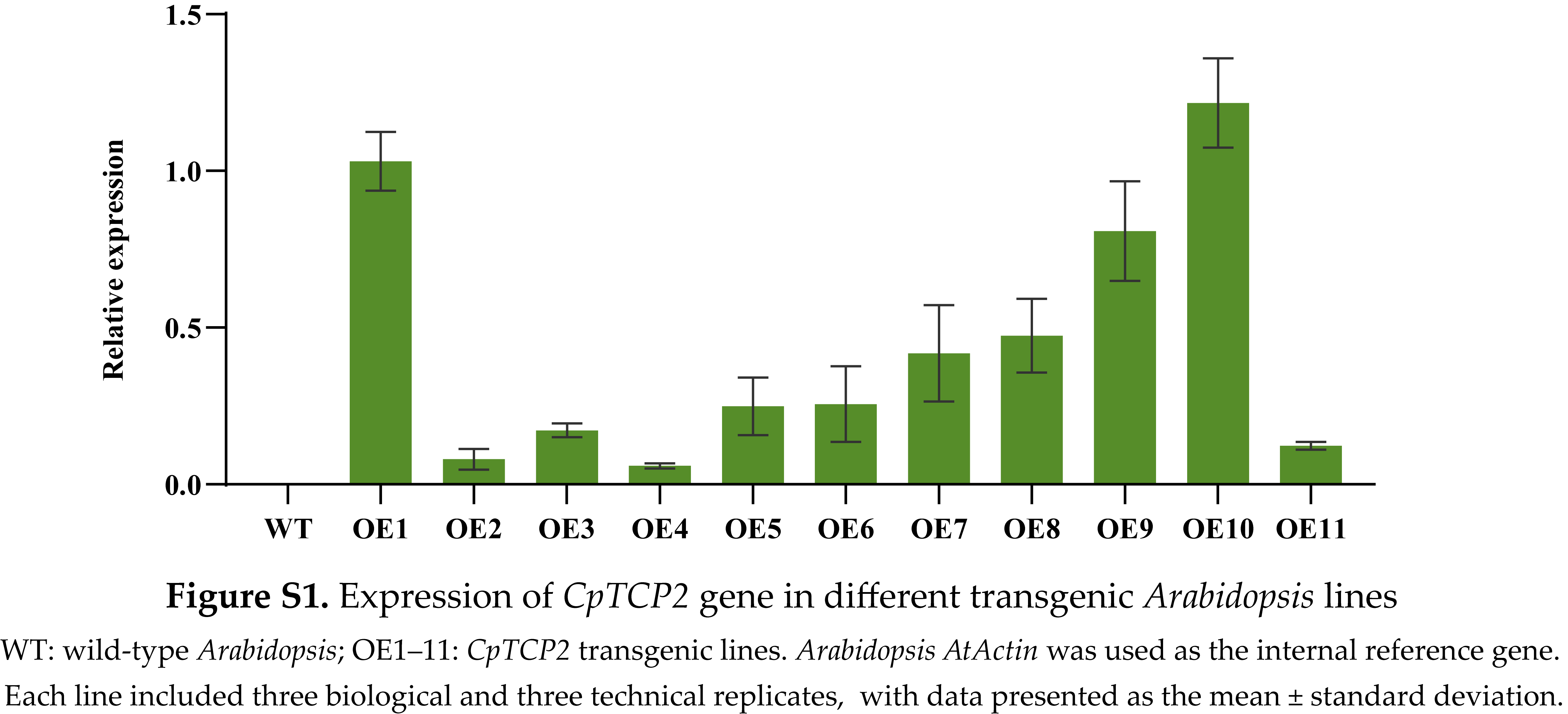

Supplement: Supplementary file 1 [file plants-14-03039-s001.zip › Supplementary Materials/Figure S1.png]
